# Personality disorders and addiction A study of 54 patients

**DOI:** 10.1192/j.eurpsy.2024.1359

**Published:** 2024-08-27

**Authors:** Y. Bensalah, L. Tbatou, M. Sabir, F. Elomari

**Affiliations:** ^1^Psychiatric hospital Ar Razi, salé, Morocco

## Abstract

**Introduction:**

Personality disorders are very often comorbid with addictions and are known to have a negative impact on the development of substance use disorder.

**Objectives:**

Evaluate the prevalence of personality disorder in patients with problematic use of psychoactive substances followed at Ar Razi hospital Determine the relationship between different personality disorders and the clinical aspects of psychoactive substance use

**Methods:**

This is a descriptive and analytical cross-sectional study carried out among 54 patients followed at Ar-Razi hospital Salé in Morocco for problematic use of psychoactive substances from June 1 to September 15, 2023 (Diagnosis assessed by DSM 5) Data collection was done using a questionnaire including clinical and socio-demographic characteristics and data on addiction to psychoactive and behavioral substances. The psychometric scale used to assess personality disorder: Personality Disorder Questionnaire (PDQ-4+)

**Results:**

We recruited 54 patients with age ranges from 18 to 45 years, with a male predominance. The average age at the start of psychoactive substance use was 15 years. Tobacco is the most used substance followed by cannabis Antisocial, histrionic and borderline personality disorders were the most common in our population. There was a statistically significant difference between specific personality disorder and the presence of severe psychoactive substance use disorder

**Conclusions:**

The frequency of personality disorders is high among subjects with problematic use of psychoactive substances. It is necessary to take care of them simultaneously (integrated care) in order to improve the prognosis

**Disclosure of Interest:**

None Declared

